# Reports of Clinical Cases

**Published:** 1890-07

**Authors:** G. M. Pease

**Affiliations:** San Francisco, Cal.


### REPORTS OF CLINICAL CASES.

G. M. Pease, M. D., San Francisco, Cal.

Read before the California State Homœopathic Medical Society, May 14th, 1890.

Pneumonia.—Mrs. Y., a very fleshy woman of about thirty years, was taken with a severe chill during the night previous to my visit. When seen she was breathing rapidly—forty-eight respirations per minute—had a pulse of one hundred and forty; frequent short, hacking cough; considerable thirst for moderate quantities; flushed face, and great restlessness, each change of position giving relief for a moment.

Rhus-tox.$^{200}$ was prepared in water and a dose given at once, to be repeated in one hour, but afterward not oftener than once in two hours. As soon as she was aware of improvement no more of the remedy was to be taken unless getting worse. The next day she was found with normal pulse and respiration. She had taken but two doses, as before the time for the third she felt easier. There was no cough or expectoration following, and on the next day she resumed her household duties. No auscultation was made, but from the symptoms manifested there could be little doubt as to the nature of the trouble.

Pleuro-Pneumonia.—Mrs. M. had been treated for la grippe by her allopathic physician, and after a week or more of steadily getting worse, her doctor sent word that he was too sick to attend her. Homœopathy had been tried for the children, and in the emergency she concluded to risk it herself. When seen she was sitting propped up in bed, breathing rapidly, forty-two respirations per minute, and screaming with nearly every breath on account of the cutting pains in the left side; her pulse was irregular but about one hundred and sixty per minute, and temperature one hundred and three and four-fifths. Frequent coughing was attended with extreme pain. Thirst great, could hardly get enough water. A few hours previously there had been some vomiting. A physical examination was not possible owing to a plaster on the back, a flaxseed poultice on each side, and flannel saturated with tar, turpentine, and oil on the chest.

The applications were at once discontinued, and Bryonia$^{200}$ given in water every two hours. She was first seen about five P. M. A much more comfortable night than the previous one was passed.

The following afternoon showed great change; temperature, one hundred and one-half; pulse, one hundred and ten; very little thirst; cough loose, some expectoration, a portion of which had been rusty-colored, as reported by her husband. Pains much less. No change was made in the remedy, except to lengthen the intervals to four hours between doses.

She steadily improved, and the case was dismissed on the sixth visit. Sac-lac. was given after the third day.

DIPHTHERIA.—On Tuesday, Mr. M. was feeling poorly, gradually getting worse, so that by noon he was obliged to give up his business. Felt feverish and weak, his knees were so weak that they ached. Throat was a little painful on swallowing, worse on left side. He retired early, and upon getting into bed had a severe chill lasting for nearly half an hour. He was inclined to be restless, but compelled to keep pretty quiet because the least movement toward a cool place would bring a return of the chill. There was no thirst. Upon inspection the throat looked simply a little inflamed. He was not subject to sore throat. About nine P. M. a single dose of Rhus-tox.$^{200}$ was given.

In the morning of Wednesday, less fever, but very weak. Left tonsil covered with a large patch of membrane, the right with a small one. He could taste and smell his fœtid breath. No medicine. Thursday the membrane was gone from the right side and was much less on the left. No fever, breath less fœtid, was up and dressed in his room. Thursday evening, membrane still less in extent and very thin. Friday, membrane entirely gone, no pain in the throat, appetite returning, felt as if he had been sick a month. Saturday, out about his business. Only one dose of medicine was given, no washes, gargles, or local treatment of any sort was employed.

Chronic Diarrhœa.—S. has had chronic diarrhœa for two or three years, at times better, but never having less than four or five discharges daily.

In many points the description might agree with any one of a dozen remedies. Close questioning, however, elicited two very important guiding symptoms. About the middle of the forenoon, or from ten to eleven A. M., he had an empty, unpleasant feeling at the stomach, which compelled him to eat something. When in bed his feet were hot to the extent that he was constantly moving them to find a cool place, even sometimes putting them from under the covers.

Four doses of Sulphur$^{200}$ were given him, to be taken during the following twenty-four hours. In a week he reported that there had been an almost instant change of his symptoms, and he had craved and eaten meat, to which he had previously had a great aversion. This symptom I had not learned on his first call. In another week he reported himself as well, could eat anything his fancy dictated, and there was no more diarrhœa, the only symptom remaining, as far as he could see, was the occasional empty feeling at the stomach about eleven A. M.

